# Non-*Apis* Bee Exposure Workshop: Industry Participants’ View

**DOI:** 10.1093/ee/nvy138

**Published:** 2018-12-04

**Authors:** Silvia Hinarejos, John Abbott, Anne Alix, Sharma Bibek, Ana Cabrera, Timothy Joseph, Bridget O’Neill, Rajwinder Singh, Helen Thompson

**Affiliations:** 1 Valent U.S.A. LLC, Dublin, CA; 2 Syngenta Crop Protection LLC, Greensboro, NC; 3 Corteva Agriscience, The Agriculture Division of DowDupont, Milton, Milton Park, Abingdon, Oxfordshire, United Kingdom; 4 FMC Corp., Ewing, NJ; 5 Bayer Crop Science LP, Research Triangle Park, NC; 7 Corteva Agriscience, The Agriculture Division of DowDupont, Indianapolis, IN; 8 BASF Corp., Research Triangle Park, NC; 9 Syngenta, Jealott’s Hill International Research Station, Bracknell, Berks, United Kingdom

The use of pesticides, as formulated products, and their active ingredients are regulated to ensure that products are effective in crop protection, and at the same time, safe for humans and the environment. Because nontarget organisms, such as bees, can be exposed to pesticides, a comprehensive body of legislation has been established in multiple countries to evaluate the safety of their uses. According to those regulations, pesticides should be authorized only in ways that do not pose an unacceptable risk to bees. For this purpose, each pesticide must pass a bee risk assessment during the authorization process (also called registration) before it can be used.

The crop protection industry develops tools for farmers, so that they can effectively protect their crops from pests and diseases while avoiding unintentional effects to the environment, such as causing harm to pollinators and the availability of healthy bees. Industry must comply with pesticide regulations applicable in each country or region while is also engaging in stewardship programs with multiple stakeholders, such as farmers, researchers, government agencies, beekeepers, and conservation groups, to promote good pesticide management practices and bee health.

Risk assessment procedures established by regulatory agencies are designed to inform risk management decisions, ensuring that approved uses of pesticides are compatible with the protection of bees. If a potential risk is identified, appropriate risk mitigation measures are implemented. Regulatory agencies such as the United States Environmental Protection Agency (USEPA), Health Canada Pest Management Regulatory Agency (PMRA), California Department of Pesticide Regulation (CDPR), European Food Safety Authority (EFSA), Brazilian Institute of the Environment and Renewable Natural Resources (IBAMA), and Australian Pesticides and Veterinary Medicines Authority (APVMA) have defined protection goals related to bees. They generally include the maintenance of pollination services and hive products (e.g., honey, wax, propolis), and bee biodiversity (EC 2009; USEPA et al. 2012, 2014; APVMA 2017; Cham et al. 2017). Although these protection goals are considered protective for all bee species, the means to achieve them across *Apis* and non-*Apis* bees in the regulatory context may differ.

To date, most pesticide-regulatory assessments rely on the honey bee as a surrogate species for the wide range of bee species associated with agricultural and horticultural crops worldwide. The Western honey bee (*Apis mellifera* L.; Hymenoptera: Apidae) was selected by regulatory agencies as a suitable model due to its availability and ability to thrive under laboratory testing conditions. It is assumed that data for individual honey bee adults or larvae, as well as honey bee colonies, can provide relevant information on the potential effects and exposure of pesticides to non-*Apis* species (solitary bees, bumble bees, and stingless bees). However, uncertainties exist regarding *the extent* to which pesticide exposure data for honey bees can be considered protective for non-*Apis* bees. Honey bees and non-*Apis* bees differ due to their respective life-history traits (i.e., body size, sociality, flight season, voltinism, floral specialization, and nesting behavior), which may result in different ecological impacts from pesticide use (Tasei 2002; Brittain et al. 2011).

There are fewer published efforts defining pesticide exposures to solitary and social non-*Apis* bee species than there are for honey bees. In terms of exposure, there is a lack of documented scenarios to account for the differences between *Apis* and non-*Apis* life-history characteristics, creating uncertainty within the regulatory community as to whether honey bee exposure estimates are protective for other bee species as regards exposure and related risks. On this basis, and under the initiative of the Pollinator Research Task Force, an international workshop was held on 10–12 January 2017 in Washington, DC. The workshop was organized as a tripartite effort among regulatory agencies, academia, and the crop protection industry. Forty bee researchers and risk assessors from 10 different countries gathered to discuss the state of science on pesticide exposure to non-*Apis* bees. The objectives of the workshop were to identify potential exposure routes to non-*Apis* bees (solitary bees, bumble bees, and stingless bees) and compare them to routes of exposure currently parameterized for honey bees in different regulatory risk assessment schemes. The exposure routes were classified as primary or secondary, based on the participants’ collective expert judgment. Considering the importance and the sensitive nature of pesticide risks to bee health, this meeting also offered the opportunity to build trust and reliability in a pollinator risk assessment that may not be familiar by all participants, but which is very often under scrutiny by public opinion. Participants were invited to reduce uncertainties and ambiguities, clarify facts, and identify research needs that can better inform future bee risk assessment and risk management decisions.

Further discussions on non-*Apis* pesticide exposure risks occur in a series of accompanying articles, but one of the conclusions of the workshop was that the dietary exposure through consumption of pollen and nectar by adults and larvae is the most important exposure route for honey bees and non-*Apis* bees. Here, we provide an additional point of view from industry scientists (users of the pollinator risk assessments) of the multiple conservative exposure assumptions in the current honey bee risk assessment, and whether they may adequately cover the potential dietary exposure for individual non-*Apis* bees.

In the honey bee risk assessment methodology, a tiered approach is used, where tier 1 is based on laboratory toxicity data, aiming at measuring the intrinsic toxicity of a substance to adult or developing stages, and modeled exposure estimates to individual honey bees. Tier 1 is intentionally very conservative and serves as a screening method for determining whether additional higher-tier testing at the colony level is needed to inform the risk assessment. Consequently, in tier 1 assessments, pesticide exposures are estimated based on high-end daily pollen and nectar consumption rates of each honey bee caste.

For adults, the screening method relies upon nectar-foraging bees, which consume more than other adult worker bees (Rortais et al. 2005). This consumption rate is comparable to the consumption rates of adult drones and will be protective for adult queens as well. Although the queen consumes more food daily than adult workers and drones, she consumes only royal jelly, which, if pesticide residues do exist, contains orders of magnitude less pesticide residues than that consumed by workers (USEPA et al. 2012; Böhme et al. 2017). Based on overly conservative energy demands of foraging flights (Rortais et al. 2005), coupled with a rather low estimate for sugar content in nectar of 30% across all crops in the United States, Canada, and Brazil (15% in Europe), the agencies assume that an individual forager bee will consume 292 mg nectar per day (853 mg in Europe), the highest consumption compared to other life stages. On the basis of an exposure of adults of three times bodyweight of nectar per day (128 mg average body weight according to Johansen and Mayer,1990), a rather low estimate for sugar content in nectar of 30%, no other source of food, and a continuous foraging for 10 h/d with 80% of time flying, the risk assessment assumes 150 flights/d (Rortais et al. 2005). Data from tagged bees in real world conditions demonstrate over a period of days bees forage on canola 2–3 times per day (Thompson et al. 2016); even 20–30 foraging flights would mean this is a fivefold overestimate.

For larvae, the screening method relies upon consumption data for a 5-d old larva, which consumes the most food compared to other larval instars. Exposures through consumption of royal jelly and brood food are not explicitly accounted for in the tier 1 exposure method. The larval consumption rate is estimated to be a continuous exposure of larvae of 120 mg nectar and 3.6 mg pollen per day throughout its development, from hatching to pupation. Data in literature show that from day 3 brood food contains limited amounts of nectar and is free or contains traces of pollen (Lucchetti et al. 2018). The proposed method used in risk assessment is expected to be conservative and also potentially protective for larvae of solitary bees and bumble bees that feed on a mix of pollen and nectar, for two primary reasons. First, because the daily food consumption rate proposed for larvae is much larger than the consumption rate of honey bee brood food, which is 30 mg over 3 d (US EPA 2012). Second, because pesticide concentrations in pollen and nectar are expected to be greater than those in royal jelly and brood food (Böhme et al. 2017).

There are other lines of conservatism in the tier 1 exposure assessment methodology. Even though honey bees can forage over large distances, the regulatory agencies assume for a risk assessment that bees will exclusively forage and provision hives from the treated crop, i.e., there will be no dilution with nectar or pollen from untreated plants. Most crops pollen analysis suggests less than 50% will be sourced from a single crop in an agricultural landscape (Requier et al. 2015), thus assuming 100% is at least a twofold overestimate. The assumption in U.S. assessment (US EPA et al. 2014) is that pesticides do not dissipate or degrade over time. EFSA risk assessment (EFSA 2013) assumes a half-life (DT_50_) of 10 d as a default value for most insecticides based on first-order kinetics (i.e., the residues will be 50% after 10 d). Dissipation and degradation of residues from plant material may be actually more rapid thanks to physical parameters like volatilization or wash-off, physicochemical factors like photolysis, abiotic chemical degradation as well as biotic metabolization and dilution due to plant growth (Willis and McDowell 1987; EFSA 2009). In the absence of compound-specific data of residues in pollen and nectar, the EPA assessment for foliar applications assumes that residue levels in nectar equivalent to 98-mg active ingredient/Kg per an application rate of 1 Kg a.i./ha, although a residue data set used in the EFSA assessment (derived from multiple chemicals, plant matrices, and study sources) shows a median and a maximum in nectar of 2.9 mg a.i./Kg and 20.7 mg a.i./Kg per 1 Kg a.i./ha, respectively. Consequently, the EPA assumption is a fourfold overestimate even for the maximum. In cases where tier 1 exposures are found to exceed the level of concern, exposure estimates can be revisited or “refined” with compound-specific empirical (measured) residue data in pollen and nectar from treated crops following the worst-case label recommendations. In such cases, to account for possible site-to-site and year-to-year variability, the agencies require data from multiple sites located in different geographical regions and in different years, yielding studies with high replication. Even if large data sets are available for a single crop, the agencies tend to select the maximum concentrations resulting from these residue studies in their refined exposure assessments. The use of maximum concentrations is considered conservative when assessing chronic risks, as honey bees forage across different crops, which vary in residues both in space and time (i.e., they don’t store and use only the forage from a single day). In addition, these maximum residue values are compared to toxicity endpoints from laboratory studies, where honey bees are exposed to a repeated (chronic) consistent level of exposure over time.

Overall, the current honey bee dietary exposure assessment paradigm used by various regulatory agencies can be considered very conservative. The exposure for chronic honey bee risk is typically overestimated ca. 10- to 20-fold; for acute risk, the degree of overestimation may be less but still conservative. Based upon the current knowledge, there was agreement by participants in the workshop that it is appropriate to use honey bees to assess effects and exposure of pesticides to individual bees. However, important research gaps such as the metabolic needs of non-*Apis* bees to power flight (among other activities) were identified. Understanding consumption rates for adults and larvae across non-*Apis* bee species should be the focus of research in future that will help to better inform whether there are major differences with the dietary estimates used in the current honey bee risk assessments. Although further developed in accompanying articles, we also recognize the need for further research to determine if the unaccounted routes of exposure in the honey bee model are significant or not for non-*Apis* bees species, especially those related to the use of nesting materials. Although over the course of workshop, some model approaches to obtain exposure level estimates were proposed, future research quantifying residue levels of these materials in real world conditions will help to better inform risk assessments, and potentially calibrate those models.

Finally, there are many clear advantages in using honey bees to evaluate pesticide exposure and effects (Table 1). Honey bees are 1) readily available in different regions; 2) less genetically diverse; 3) multivoltine (i.e., have several brood cycles per year); 4) relatively easy to rear and maintain, and 5) generalist foragers (i.e., forage on many flower types in crop fields and bordering landscapes). Test methods for regulatory use are available to assess effects (toxicity) in laboratory, as well as in semifield, and field conditions. Exposure to pesticide residues in pollen and nectar is feasible, by sampling and chemically measuring these matrices from treated plants, individual honey bees returning to the hive, or in the hive stores. Many of these advantages are not applicable for other commercially managed non-*Apis* bee species, making them less suitable surrogates either for assessing effects or exposure. Although not the focus of this workshop, regulatory toxicity study (“test” used synonymously here) protocols to evaluate the effects of pesticides on non-*Apis* bees are also being developed under the auspices of international organizations such as the Organization for Economic Co-operation and Development (OECD) and the International Commission for Plant–Pollinator Relationships (ICP-PR), where industry is working in collaboration with academia and regulatory agencies. The purpose of these studies is to determine the intrinsic toxicity of a substance to adults or immature stages of non-*Apis* bee species in laboratory conditions, with the objective to be able to compare the relative sensitivity of the species to that of the honey bee, and ultimately help determine if the honey bee is protective of other bee species. When developing these tests, some modifications of existing honey bee protocols may be necessary to account for fundamental biological and behavioral differences of non-*Apis* bees, e.g., the acceptance of artificial feeders by solitary bee adults. Unlike honey bees, solitary bees do not use trophallaxis to distribute information about new nectar sources, and entirely rely on floral cues to locate and discriminate food resources. Consequently, unlike honey bees, when confined to small laboratory cages solitary bee adults do not readily feed from an artificial feeder such as a syringe or a feeding tube (Ladurner et al. 2003, 2005a, 2005b). Some technical challenges have been identified (Spurgeon et al. 2016), some proposals have been made to standardize test protocols (e.g., Hinarejos et al. 2015), and further optimizations are still under development by the ICP-PR non-*Apis* bees working group. However, before tests with non-*Apis* bees are potentially included in any regulatory framework, a workable and well-vetted risk assessment process must be available, including valid protocols for refining screening-level assessments. By using validated protocols, methods are assumed by the regulatory community to be sufficiently reliable, repeatable, and reproducible by different laboratories.

**Table 1.** Surrogacy characteristics of honey bees (*A. mellifera*) *vs.* non-*Apis* bees

Surrogacy criteria	Honey bees	Non-*Apis* bees
Biology well known	Yes	Only for a few species
Readily available	Yes	Only few species managed and commercially available
Multivoltine	Yes	Only a few species, several univoltine
Genetically homogenous	Yes^*a*^	Different species in different regions
Easy to work with in laboratory	Yes	No, or unknown, research work in progress
Easy reproduction conditions	Yes	No, or unknown, research work in progress
Well-documented husbandry needs	Yes	No, only a few exceptions
Measured effects and exposure protocols available	Yes	Less readily available
Known routes and levels of exposure	Yes	Non-Apis bee exposure workshop goal

^*a*^Honey bees are considered in this comparison more genetically homogeneous compared to non-*Apis* bee species due to their domestication. However, honey bee queens mate with multiple drones thus a colony has numerous patrilines and ideally, relatively high genetic diversity. The genetic diversity is increased when comparing multiple colonies.
